# A Non-Invasive Thermal Drift Compensation Technique Applied to a Spin-Valve Magnetoresistive Current Sensor

**DOI:** 10.3390/s110302447

**Published:** 2011-02-25

**Authors:** Jaime Sánchez Moreno, Diego Ramírez Muñoz, Susana Cardoso, Silvia Casans Berga, Asunción Edith Navarro Antón, Paulo Jorge Peixeiro de Freitas

**Affiliations:** 1 Department of Electronic Engineering, University of Valencia, C/Doctor Moliner, 50, 46100-Burjassot, Spain; E-Mails: jaime.sanchez@uv.es (J.S.M.); silvia.casans@uv.es (S.C.B.); edith.navarro@uv.es (A.E.N.A.); 2 Instituto de Sistemas e Computadores—Microsistemas e Nanotecnologias (INESC-MN) and Institute for Nanosciences and Nanotechnologies, R. Alves Redol 9, 1000-029 Lisbon, Portugal; E-Mails: sfreitas@inesc-mn.pt (S.C.); pfreitas@inesc-mn.pt (P.J.P.F.)

**Keywords:** electrical current measurement, magnetoresistance sensor, spin-valve sensor, temperature compensation

## Abstract

A compensation method for the sensitivity drift of a magnetoresistive (MR) Wheatstone bridge current sensor is proposed. The technique was carried out by placing a ruthenium temperature sensor and the MR sensor to be compensated inside a generalized impedance converter circuit (GIC). No internal modification of the sensor bridge arms is required so that the circuit is capable of compensating practical industrial sensors. The method is based on the temperature modulation of the current supplied to the bridge, which improves previous solutions based on constant current compensation. Experimental results are shown using a microfabricated spin-valve MR current sensor. The temperature compensation has been solved in the interval from 0 °C to 70 °C measuring currents from −10 A to +10 A.

## 1. Introduction

Advances in the solid-state sensor manufacturing industries have allowed a significant improvement in the miniaturization, consumption and cost of instrumentation and measurement systems. The above benefits have been reached using semiconductors and transition metals as reference materials. However, the temperature dependence of their physical properties demands the use of specific compensation techniques, especially where the measurand of interest will not be the temperature (pressure, vibration, electrical current, *etc.*). In this way there are various and different efforts that have been dedicated to the reduction of the sensors temperature dependence. There are two main groups in which the temperature compensation techniques could be classified: hardware compensation and digital processing compensation methods. In [1] different hardware thermal compensation methods dedicated to a piezoresistive sensor are presented. Particularly, it is required to have complete accessibility to the sensor Wheatstone bridge terminals or the use of active components (like bipolar transistors). The temperature compensation of the fluid to be measured is of special relevance in a well-done flowmeter design. Following this goal, various solutions are proposed in [2,3] where some mathematical conditions must be satisfied or where two Wheatstone bridges were needed. In the power electronics field the techniques described in [4,5] are interesting. In [4], current sensing was done by temperature compensation of the inaccuracy caused by the inductor parasitic ohmic resistance temperature dependence. The compensation was successful but complex to satisfy in its mathematical requirements. The work described in [5] was based in a frequency domain compensation procedure and was used in a low voltage power supply situation.

Digital processing algorithms have been applied reducing the thermal dependence in piezoresistive sensors [6], load cells [7] or in pressure capacitive sensors [8]. These techniques require an additional processing hardware based in digital intelligence (computer, microcontroller or digital signal processor) and dedicated software. All of them are, in a large number of industrial situations, difficult to apply.

In this work the thermal drift of a microfabricated current sensor is reduced by the use of a simple hardware compensation method. This represents an improvement of a technique that was first developed in [9]. The sensor is a Wheatstone bridge implemented by four magnetoresistances of the spin-valve type [10]. The compensation is solved using a resistive temperature sensor by means of a simple procedure because no equilibrium Wheatstone bridge condition is needed, nor two bridge units or full access to the bridge terminals are required. The magnetoresistive current sensing technology has been successfully applied in power converters [11] or in instrumentation and measurement area (current probes design) [12]. In the present work the temperature compensation has been solved in the temperature interval from 0 °C to 70 °C measuring currents from −10 A to +10 A.

## 2. Compensation Method

**Figure 1.**
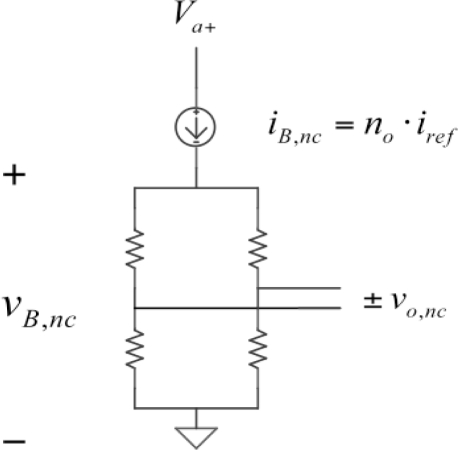
Variables definition in the Wheatstone bridge before compensation.

A constant current driven Wheatstone bridge sensor is used to apply the compensation method (Figure 1). The driving sensor current source was described in [13,14] and is based in a Generalized Impedance Converter circuit (GIC). The choice of a constant current source as the sensor bridge power supply was based on previous works that show a remarkable reduction in the temperature coefficient of the bridge sensitivity [15–18].

If a current sensor is considered, its non-compensated output, *v_o,nc_* could be given by:

(1) $$v_{o,\mathrm{nc}}\;(t)=S(t)\cdot i+v_{o,\mathrm{off}}$$

being *i* the current to be sensed (measured in amps), *v_o,off_* the sensor output at zero input current (in mV), *t* the temperature (in °C) and *S(t)*, the sensor sensitivity (in mV/A). The output offset could not be considered if this has been compensated previously by calibration. As equation (1) shows, a change in temperature will produce a change in the sensor sensitivity generating an output sensor variation, but without any change in the current *i* to be measured.

Let S¯≡SvB,nc be the sensor sensitivity normalized to bridge voltage, *v_B,nc_*. If the sensor bridge is driven by a constant current *i_B,nc_* the voltage drop across it will be:

(2) $$v_{B,\mathrm{nc}}\;(t)=i_{B,\mathrm{nc}}\cdot R_{B}\;(t)=n_{o}\cdot i_{\mathrm{ref}}\cdot R_{B}\;(t)$$

where *R_B_* is the equivalent bridge resistance. The constant current *i_B,nc_* in equation (2) is supplied by the GIC circuit and it is equal to the product of the constant *n_o_* times the GIC input reference current *i_ref_*, [13,14]. As a consequence, the non-compensated sensor bridge output *v_o,nc_* will be given by:

(3) $$v_{o,\mathrm{nc}}\;(t)=\bar{S}(t)\cdot v_{B,\mathrm{nc}}\;(t)\cdot i=\bar{S}(t)\cdot i_{B,\mathrm{nc}}\cdot R_{B}\;(t)\cdot i=\bar{S}(t)\cdot n_{o}\cdot i_{\mathrm{ref}}\cdot R_{B}\;(t)\cdot t$$

Equation (3) shows that the temperature drift of the sensor output will be provided by both normalized sensitivity and bridge equivalent resistance temperature dependences. It is assumed that before compensation *i_ref_* is a good reference current and it is not affected by the temperature.

The main purpose of the compensation method is to place enough temperature dependence in *i_ref_* to compensate the drift in *v_o,nc_(t)* caused by *S̄*(*t*) and *R_B_(t)*. As a result the compensated sensor output, *v_o,c_(t)* will have no temperature dependence:

(4) $$\frac{{\mathrm{dv}}_{o,c}\;(t)}{\mathrm{dt}}=0.$$

**Figure 2.**
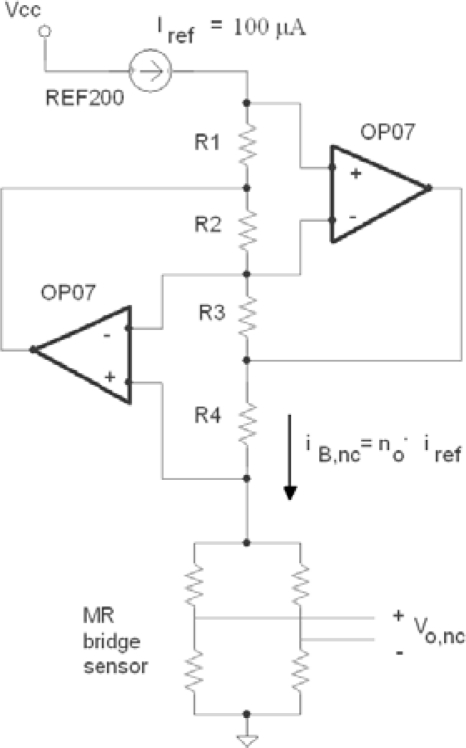
Driving the MR current sensor by a constant current source using a GIC circuit.

Figure 2 depicts how a practical GIC circuit supplies to the bridge sensor a current *i_B_* proportional to a 100 μA reference current *i_ref_*. The gain factor *n_o_* depends on GIC resistances *R1* to *R4*, a proper replacement of one of them by a series association of a temperature sensor *R_s_(t)* and a constant resistance *R* will lead to accomplish equation (4). The final objective of the compensation method will be to find a properly selected R resistance value.

The gain factor is given by:

(5) $$n_{o}\equiv \frac{R_{1}\cdot R_{3}}{R_{2}\cdot R_{4}}$$

and four possibilities could be considered to enter a temperature dependence on it depending on whether the series association is placed [equations (6) to (9)]:

(6) $$R_{1}=R+R_{s}\;(t)\to n(t)=\frac{\left[R+R_{s}\;(t)\right]\cdot R_{3}}{R_{2}\cdot R_{4}}$$

(7) $$R_{3}=R+R_{s}\;(t)\to n(t)=\frac{R_{1}\cdot \left[R+R_{s}\;(t)\right]}{R_{2}\cdot R_{4}}$$

(8) $$R_{2}=R+R_{s}\;(t)\to n(t)=\frac{R_{1}\cdot R_{3}}{\left[R+R_{s}\;(t)\right]\cdot R_{4}}$$

(9) $$R_{4}=R+R_{s}\;(t)\to n(t)=\frac{R_{1}\cdot R_{3}}{R_{2}\cdot \left[R+R_{s}\;(t)\right]}$$

With a temperature dependence in the gain factor *n(t)*, from equation (3) the compensated bridge output voltage can be rewritten as:

(10) $$v_{o,c}\;(t)=\bar{S}(t)\cdot n(t)\cdot i_{\mathrm{ref}}\cdot R_{B}\;(t)\cdot i=\frac{S(t)}{n_{o}\cdot i_{\mathrm{ref}}\cdot R_{B}\;(t)}\cdot n(t)\cdot i_{\mathrm{ref}}\cdot R_{B}\;(t)\cdot i$$

As a consequence of this, in order to compensate the bridge output voltage *v_o,c_(t)* only the sensor sensitivity temperature coefficient, *S*(*t*) is needed:

(11) $$v_{o,c}\;(t)=S(t)\cdot \frac{n(t)}{n_{o}}\cdot i$$

Equation (11) states how the temperature drift in sensitivity could be modulated by the gain factor drift to obtain an effective compensation in the sensor output voltage. Applying natural logarithms in both terms of equation (11), differentiating and taking derivatives with respect to the temperature it is possible to obtain:

(12) $$\frac{{\mathrm{dv}}_{o,c}\;(t)/\mathrm{dt}}{v_{o,c}\;(t)}=\frac{\mathrm{dS}(t)/\mathrm{dt}}{S(t)}+\frac{\mathrm{dn}(t)/\mathrm{dt}}{n(t)}$$

and in a more compact form:

(13) $$\mathrm{TC}(v_{o,c})=\mathrm{TC}(S)+\mathrm{TC}(n)$$

where *TC* denotes temperature coefficient of the quantity to be considered. In order to have *TC(v_o,c_*) = 0, the value of the resistance *R* will be obtained from the equation:

(14) TC(n)=−TC(S)

Depending on the position of the temperature sensor *R_s_(t)* inside the GIC circuit two different expressions for resistance *R* could be obtained. If series association *R_s_(t)+R* plays the role of the resistances *R1* or *R3* [equations (6) or (7)] the equation (14) could be rewritten as:

(15) $$\frac{1}{1+\frac{R}{R_{s}\;(t)}}\cdot \mathrm{TC}(R_{s})=-\mathrm{TC}\;(S)$$

On the other hand, if the association *R_s_(t)+R* is placed in resistances *R2* or *R4* [equations (8) or (9)] equation (14) will give to:

(16) $$\frac{1}{1+\frac{R}{R_{s}\;(t)}}\cdot \mathrm{TC}(R_{s})=\mathrm{TC}\;(S)$$

From equations (15) and (16) two expressions of resistance *R* could be obtained:

(17) $$\mathrm{if}\left\{\begin{array}{c} R_{1}\\ \mathrm{or}\\ R_{3} \end{array}\right\}=R+R_{s}\;(t)\to R=-R_{\mathrm{so}}\cdot \left(\frac{\mathrm{TC}(R_{s})}{\mathrm{TC}(S)}+1\right)$$

(18) $$\mathrm{if}\left\{\begin{array}{c} R_{2}\\ \mathrm{or}\\ R_{4} \end{array}\right\}=R+R_{s}\;(t)\to R=R_{\mathrm{so}}\cdot \left(\frac{\mathrm{TC}(R_{s})}{\mathrm{TC}(S)}-1\right)$$

where *R_so_* is the value of the resistance sensor *R_s_(t)* at the temperature *t_o_* where the compensation is specified. Defining the compensation factor *m* in the form of *m≡TC(R_s_)/TC(S)* different conclusions could be considered:

- if *m* < −1: equation (17) supplies a positive value of resistance *R* and equation (18) a negative one.
- if *m* > 1: equation (17) supplies a negative value of resistance *R* and equation (18) a positive one.
- if −1 < *m* < 1: both equations (17) and (18) will give a negative *R* value.

As a consequence of this and considering only passive resistances, the compensation method will be possible and effective if:

(19) $$\mathrm{TC}(R_{s})>\mathrm{TC}(S)\;\;\;\mathrm{or}\;\;\;\mathrm{TC}(R_{s})<-\mathrm{TC}(S)$$

The above expressions mean that │*TC(R_s_)*│>│*TC(S)*│ independently of the relationship between the sign of the temperature coefficients.

Table I summarizes the resistance selection criteria to obtain the proper temperature drift compensation of the sensor bridge.

## 3. Experimental Results

In this section a MR electrical current sensor is compensated with a ruthenium based temperature sensor. Both sensors were fully microfabricated in the same substrate at the INESC-MN facilities (Lisbon). The temperature compensation sensor was integrated in the same microfabrication process as the spin-valve MR current sensor. This fact allows a close temperature matching between both sensors. Ruthenium is more useful than platinum as the material to implement the temperature sensor because its fabrication process is more compatible with spin-valve processes, as the material is often used in the spin-valve stack by several groups in synthetic antiferromagnetic layers, see for example [19]. The ruthenium target is included in the same deposition tool used for spin valve deposition and does not require the change of the deposition infrastructure configuration needed by the platinum. In spite of its lower sensitivity (0.16 %/°C) compared to platinum (0.36 %/°C) both materials belong to the same group of metals sharing good temperature linearity. Additionally ruthenium has lower fabrication costs than platinum.

The electrical current sensor is configured as a full Wheatstone bridge. Four active 3 μm × 100 μm spin-valve sensors were deposited by ion beam deposition [20] with the structure (thickness in Å): Si/Al_2_O_3_ (500)//Ta (20)/NiFe (30)/MnIr (60)/CoFe (30)/Cu (19)/CoFe (25)/NiFe (25)/Cu (10)/Ta (20)/TiWN (50), [21], where CoFe, NiFe, MnIr and TiWN stand for Co_80_Fe_20_, Ni_80_Fe_20_, Mn_77_Ir_23_ and Ti_10_W_90_N in atomic %. Free and pinned layers easy-axis are 90° oriented to get a linear characteristic. Each single spin-valve sensor element was magnetically characterized providing a minimum resistance R_min_ equal to 740.2 Ω ± 1.7 Ω and a maximum resistance R_max_ equal to 801.1 Ω ± 2.1 Ω, this mean a MR effect of 8% according to R_max_−R_min_/R_min_.

**Figure 3.**
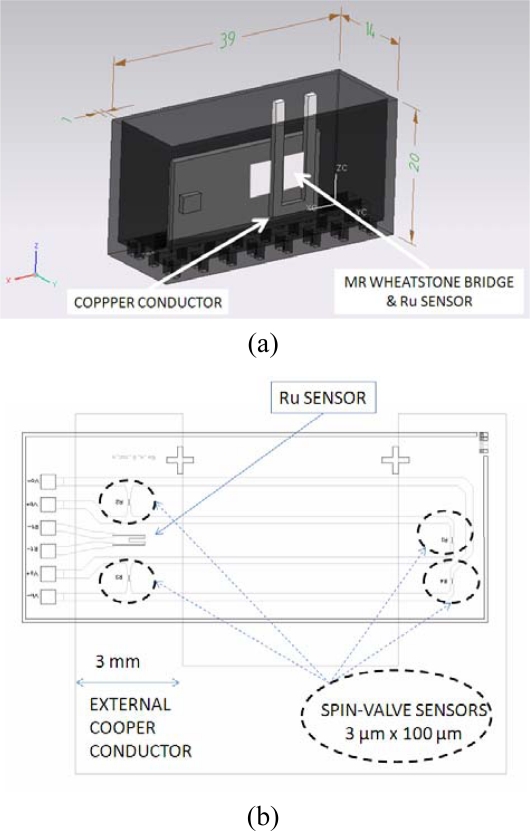
(**a**) MR electrical current sensor arrangement. (**b**) Chip layout.

The electrical current that generates the magnetic field to be measured circulated through an external copper conductor as it can be seen in Figure 3(a) (dimensions in mm). The chip layout could be shown in Figure 3(b) where the external conductor has been put on top.

Following the method described in Section 2, the MR Wheatstone sensor was biased by a constant current of 1 mA using the GIC circuit shown in Figure 2. A constant gain factor of n = 100 was designed for a reference input current of 100 μA given by the integrated circuit REF200. So the resistance values were: *R1* = 2 kΩ, *R2* = 1 kΩ, *R3* = 5 kΩ, *R4* = 1 kΩ.

**Figure 4.**
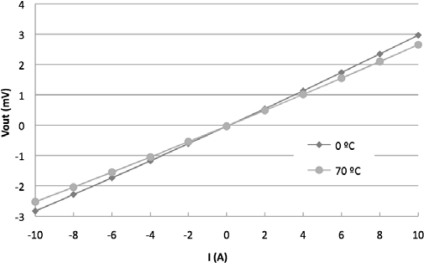
MR current sensor voltage output for a ±10 A current sweep in the extreme temperatures of the interval (1 mA bridge supply current).

**Figure 5.**
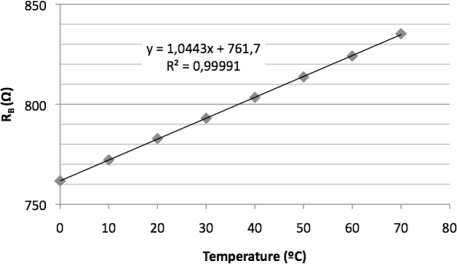
Equivalent bridge resistance value as a function of the temperature.

The MR current sensor was placed inside a climate chamber (model CH-600 from Angelantoni) that generated a controllable and stable temperature (with an accuracy of ±0.3 °C). The experimental sensitivity of the sensor was determined collecting the voltage output of the MR current sensor responding to steps of 1 A of the electrical input current that was selected within the range of ±10 A. An electrical current sweep was done at a temperature included in the interval from 0 °C to 70 °C and with increments of 10 °C. The results of these measurements corresponding to the extreme temperature values of the interval are shown in Figure 4, it could be noticed the decrease in the sensor sensitivity as the temperature increases. Taking the value 0 °C as temperature reference *t_o_* the thermal coefficient sensitivity was *TC(S)* = −0.152 %/°C. The equivalent resistance of the bridge was also measured, note that it is linear temperature dependent with opposite sign with respect to *TC(S)* (Figure 5).

**Figure 6.**
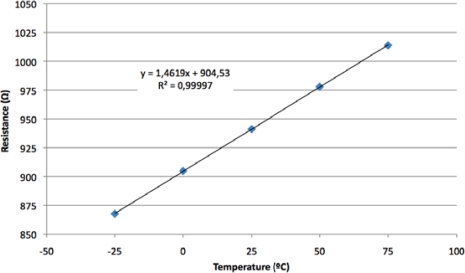
Thin film ruthenium resistance as a function of the temperature.

The sensitivity of the ruthenium thermoresistive sensor was obtained based on measurements taken with a Keithley K2700 digital multimeter. The results are depicted in Figure 6 showing the ruthenium resistance value as a function of the temperature. A linear least-squares fit was applied to the collected data in order to estimate the characteristic parameters of the Ru sensor. The main parameters obtained were a 0 °C reference resistor value of 904 Ω, a temperature sensitivity of 1.46 Ω/°C and a positive temperature coefficient *TC(R_s_)* of 0.161 %/°C. The experimental correlation coefficient was 0.998 showing the good linearity offered by the ruthenium resistance temperature detector (Figure 6).

**Figure 7.**
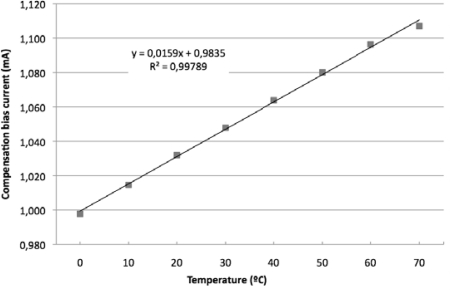
Temperature dependence of the experimental compensation current.

The GIC circuit was redesigned considering the criteria established in Table I. An obtained value of the compensation factor *m* of −1.1 will give that the series association of *R_s_(t)* and *R* will be placed in *R1* or *R3*. In the present work the resistance *R3* was replaced by the compensation network implemented by the ruthenium sensor in series with the calculated compensation resistor *R* = 91.3 *Ω*. Selecting resistances *R1* = *R2* = 2 kΩ, and *R4* = 100 Ω, the gain factor *n(t)* at 0 °C was 9.94. Figure 7 shows the current driven by the GIC circuit. It could be notice that the current source is temperature dependent and its varying level modulates, in an opposite way, the temperature variation of the bridge sensitivity. This is the fundamental purpose of the proposed method. As in [22], the objective will be to bias the bridge with a current across it that increases with temperature. This would be done biasing the bridge by a NTC resistor, a negative impedance converter (NIC) or a temperature dependent-current source as proposed. The experimental temperature coefficient of the gain factor *n* was *TC(n)* = 0.158 %/°C, in concordance with the theoretical value expected by equation (14) for a properly designed temperature compensation.

**Figure 8.**
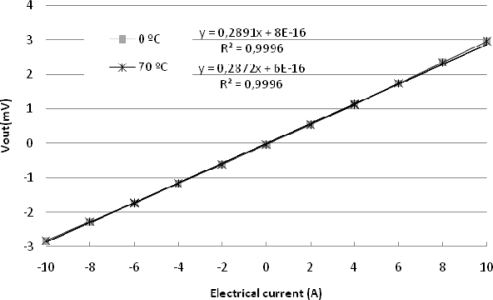
Compensated current sensor output voltage as a function of the input current under different environment temperatures.

The MR current sensor was biased with the new temperature modulated current. The comparison between Figures 4 and 8 shows that the suggested method reduces the error due to temperature drift.

**Figure 9.**
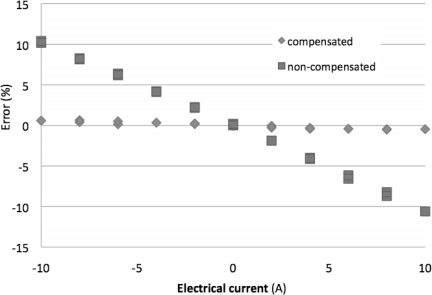
Sensor output maximum difference corresponding to the extreme temperatures (0 and 70 °C) with and without compensation.

Using temperature compensation the discrepancy between the sensor output voltage for minimum and maximum temperatures are one order of magnitude smaller than the non-compensation discrepancy (Figure 9).

**Figure 10.**
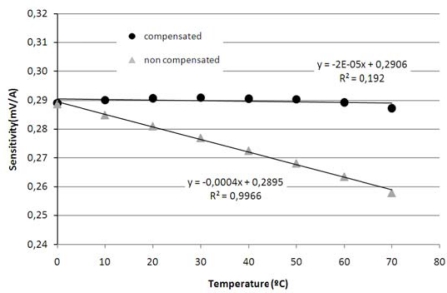
Sensitivity evolution of the non-compensated and compensated MR current sensor.

The new temperature coefficient of the sensitivity was calculated obtaining the value of *TC(S)* = 0.007 %/°C. Figure 10 verifies the performance of the compensation method demonstrating how the proposed technique is effective reducing the temperature sensitivity dependence. As it can be observed, the evolution of the compensated sensitivity is almost flat and close to the value of the sensitivity at 0 °C reference for all the analyzed temperature range. The non-compensated sensitivity was affected by temperature drift, decreasing its value as temperature rises.

## 4. Conclusions

In the present work an improved compensation method has been applied to compensate the sensitivity drift temperature coefficient of a MR current sensor. The temperature compensation has been solved in the interval from 0 °C to 70 °C measuring currents from −10 A to +10 A. The compensation technique described is useful for commercial sensors where the accessibility to its internal structure is not possible. To solve the compensation, the only requirement is that the compensation sensor must have a temperature coefficient higher than that of the sensor to compensate for. This condition is easy to find when comparing the temperature coefficient of a resistance temperature sensor with the target sensor temperature drift.

The temperature compensation sensor was integrated in the same microfabrication process as the spin-valve MR current sensor. This fact allows a close temperature matching between both sensors. Ruthenium is more useful than platinum as the material used to implement the temperature sensor because its fabrication process is more compatible with existing spin-valves processes. Ruthenium is a material used in some spin-valves fabrication processes and does not require any changes to the deposition infrastructure configuration as needed by platinum.

The proposed technique has been applied to a magnetoresistive current sensor that finds application in industrial switched-mode power supplies, power converters or electrical drives. The current source designed by the GIC circuit improves the other classical constant current sources like the Howland circuit because it does not need good matching between resistances involved in the circuit and without requiring positive feedback in the GIC circuit as it occurs in the Howland current source.

## Figures and Tables

**Table 1.** Resistance selection criteria to obtain the proper temperature drift compensation of the sensor bridge.

		**Compensation Factor, m**	
		**m≤1**	**m>1**
**Compensation sensor**	*R_s_(t)*	*R_1_ or R_3_*	*R_2_ or R_4_*
**Compensation resistance**	*R*	−*R_so_* · [*m* + 1]	*R_so_* · [*m* − 1]

## References

[1] Guo T, Shi Y, Zhang W Study on the temperature compensation method of piezoresistance silicon film sensor.

[2] Nam T, Kim S, Kim S, Park S The temperature compensation of a thermal flow sensor with a mathematical method.

[3] Sosna C, Buchner R, Lang W (2010). A temperature compensation circuit for thermal flow sensors operated in constant-temperature-difference mode. IEEE Trans. Instrum. Meas.

[4] Tsang KM, Chan WL (2009). Temperature compensated high efficiency inductor current sensor. Sensor. Actuator. A.

[5] Dallago E, Passoni M, Sassone G (2000). Lossless current sensing in low-voltage high-current dc/dc modular supplies. IEEE Trans. Ind. Electron.

[6] Snook RD, Zaft PE (1989). Fourier-analysis method for temperature compensation of a microcomputer-controlled piezoelectric crystal sulfur-dioxide sensor. Analyst.

[7] Matsuzaki T, Kohashi T Thermal error compensation method for weighing sensor.

[8] Wang W Non-linearity Estimation and Temperature Compensation of Capacitor Pressure Sensors Using Least Square Support Vector Regression.

[9] Ramírez Muñoz D, Sánchez Moreno J, Casans Berga S, Reig Escrivà C, Navarro Antón AE (2006). Temperature compensation of Wheatstone bridge magnetoresistive sensors based on generalized impedance converter with input reference current. Rev. Sci. Instrum.

[10] Freitas PP, Silva F, Oliveira NJ, Melo LV, Costa L, Almeida N (2000). Spin valve sensors. Sensor. Actuator. A.

[11] Pelegrí J, Ramírez D, Sanchis E, Navarro AE, Casans S (2000). Giant magnetoresistive sensor in conductance control of switching regulators. IEEE Trans. Magn.

[12] Sánchez-Moreno J, Ramírez D, Casans S, Cubells MªD, Reig C, Navarro AE Extending magnetoresistive AC transfer characteristic for current measurement.

[13] Ramírez-Muñoz D, Blat González A, Sánchez Moreno J, Casans Berga S, Reig C, Navarro Antón AE Series Sensor Current Loop from a Generalized Impedance Converter Circuit with Reference Current Input.

[14] Ramírez-Muñoz D, Casans-Berga S, Reig C (2005). Current loop generated from a generalized impedance converter: a new sensor signal conditioning circuit. Rev. Sci. Instrum.

[15] Petersen A (1986). The magnetoresistive sensor. A sensitive device for detecting magnetic field variations. Electron. Compon. Appl.

[16] Gakkestad J, Ohlekers P, Halbo L (1995). Compensation of sensitivity shift in piezoresistive preassure sensors using linear voltage excitation. Sensor. Actuator. A.

[17] Philips Components (1989). Data Handbook SC17.

[18] Smith CH, Schneider RW The growing role of solid-state magnetic sensing.

[19] Veloso A, Freitas PP (2000). Spin valve sensors with synthetic free and pinned layers. J. Appl. Phys.

[20] Gehanno V, Freitas PP, Veloso A, Ferrira J, Almeida B, Soasa JB, Kling A, Soares JC, da Silva MF (1999). Ion beam deposition of Mn-Ir spin valves. IEEE Trans. Magn.

[21] Veloso A, Freitas PP, Wei P, Barradas NP, Soares JC, Almeida B, Sousa JB (2000). Magnetoresistance enhancement in specular, bottompinned, MnIr spin valves with nano-oxide layers. Appl. Phys. Lett.

[22] López-Martín AJ, Osa JI, Zuza M, Carlosena A (2003). Analysis of a Negative Impedance Converter as a Temperature Compensator for Bridge Sensors. IEEE Trans. Instrum. Meas.

